# Effect of column and pile configuration and dimension on shear performance of reinforced concrete pile caps

**DOI:** 10.1038/s41598-025-05303-7

**Published:** 2025-06-20

**Authors:** Sabry Fayed, Moataz Badawi, Ali Basha, Mohamed Ghalla, Yahia Iskander, Saad A. Yehia

**Affiliations:** 1 Department of Civil Engineering, Faculty of Engineering, Kafrelsheikh University, Kafrelsheikh, Egypt; 2 Department of Civil Engineering, College of Engineering and Architecture, Umm Al-Qura University, Makkah, Saudi Arabia; 3 Civil Engineering Department, Higher Institute of Engineering and Technology, Kafrelsheikh, Egypt

**Keywords:** Reinforced concrete pile caps, Column-pile configuration, Shear performance, Finite element modeling, Bearing capacity, Engineering, Civil engineering

## Abstract

Despite extensive research on reinforced concrete (RC) pile caps, the influence of column and pile configuration and dimensions on their shear performance remains unexplored. This study investigates the structural behavior of RC pile caps through experimental and numerical analyses, focusing on how variations in column and pile geometry affect shear capacity. Two pile cap specimens (700 mm long × 300 mm wide) with heights of 250 mm (SB1) and 350 mm (SB2) were tested under shear-dominated conditions. Both were supported by two square piles (200 × 200 mm) and loaded centrally via a square column (200 × 200 mm). The study reports crack patterns, ultimate shear load, load-displacement behavior, elastic stiffness, and energy absorption capacity. A validated 3D finite element model was developed to parametrically analyze rectangular/circular columns and piles with dimensions ranging from 0.2d to d (where d = pile cap width). The findings indicate that failure modes were consistently shear-dominated and remained unaffected by variations in column or pile configuration and size. Increasing the rectangular column length from 0.2d to d enhanced the ultimate load capacity by 108% and energy absorption by 100%. Similarly, increasing the circular column diameter from 0.2d to d improved these metrics by 348% and 373%, respectively. Widening the rectangular pile from 0.2d to d resulted in a 34% increase in ultimate load capacity. Overall, the study demonstrates that larger column and pile dimensions significantly enhance shear performance, with circular configurations yielding superior improvements. These insights offer practical guidance for optimizing pile cap design.

## Introduction

A reinforced concrete pile cap is a dense RC concrete slab designed to form a structural connection between a pier or column and one or more piles. The RC pile cap is a crucial foundation element, typically designed to transfer the pressure from the column to the supporting RC piles. To optimize the performance and design of RC pile caps, understanding the behavior of individual piles and pile groups under various loading and soil conditions is essential^1–7^. Ateş and Şadoğlu^1^ investigated single pile behavior in sand through experiments and FEM analysis. Stress and strain distributions were measured using gauges. Results showed that increasing sand relative density enhances bearing capacity and reduces settlement, confirming load transfer mechanisms and validating the numerical model with ABAQUS. Hamed et al.^2^ numerically analyzed axially loaded steel pipe piles embedded in organic soil using Plaxis 3D. Varying L/D and d/D ratios, they found that load capacity increases with pile length and wall thickness. Numerical results showed strong agreement with experimental data, validating the model’s accuracy. Ates and Şadoğlu^3^ experimentally studied piled raft foundations in sand, varying pile spacing, diameter, length, and relative density. Results showed that increasing pile spacing enhances bearing capacity up to an optimum of 4D. The study provides practical design parameters for efficient foundation engineering applications.

In another study, Ateş and Şadoğlu^4^ experimentally compared the bearing capacity and settlement of raft and piled raft foundations in sandy soil. Results offer a practical reference for evaluating driven piles without costly field tests, aiding preliminary design assessments. Ateş and Şadoğlu^5^ studied vertical stress increments in piled raft foundations using experimental and numerical methods. Model tests with composite piles and steel raft in sandy soil showed that stress decreases below pile tips and reduces further with increased relative density. FEM analysis via ABAQUS confirmed results, providing insights and parameters for foundation design applications. Ateş and Şadoğlu^6^ conducted experimental tests on pile groups in sandy soil to investigate group efficiency (η), often overlooked in prior studies. Tests varied pile spacing, relative density, and L/D ratios. Contrary to traditional beliefs, group efficiency exceeded one—ranging from 1.31 to 1.66—highlighting significant effects of pile interaction and soil conditions on performance. Efthymiou and Vrettos^7^ used FEM to study vertical vibrations in piled foundations under stationary and moving harmonic loads. Results showed that pile row configuration significantly affects vibration reduction. A stationary load near the pile group accurately approximates moving load effects, simplifying vibration analysis and design in practice.

Furthermore, RC pile caps are commonly engineered to handle lateral or/and gravity loads, and overturning moments. However, significant focus in established design standards and codes^1–5^ has been dedicated to the detailed design and analysis of columns and piles. In contrast, the design of reinforced concrete (RC) pile caps has not yet been given the necessary level of attention^8,9^. Design codes recommend two main approaches for pile caps: the sectional approach and the Strut-and-Tie Method (STM)^9^. The sectional approach treats pile caps as large beams spanning piles, designed similarly to two-way slabs or shallow footings resting on soil. The beam flexure theory is used to calculate longitudinal reinforcement at the critical section, while concrete alone resists shear forces through adequate reinforcement. Recent concrete codes of practice have adopted the Strut-and-Tie Method (STM) due to its ability to capture stress flow within structures, in contrast to the sectional approach, which calculates pile cap capacity at critical sections. STM models compression stresses as struts and tensile stresses as ties. This encompasses structures with static or geometric discontinuities, such as deep beams, corbels, and beam-column joints, where the Strut-and-Tie Method (STM) has proven effective^10,11^. Unlike deep beams, deep pile caps are large concrete blocks with low longitudinal reinforcement, making them more prone to shear. Due to their width, a separate, refined three-dimensional (3D) Strut-and-Tie Method (STM) is required for their design.

Extensive experimental, analytical, and numerical studies have been carried out to date to understand the flexural and shear behavior of RC pile caps. Blévot and Frémy^12^ developed a simplified strut-and-tie model for the design of pile caps with two to four piles, illustrating load transfer through inclined concrete struts (in compression) and horizontal reinforcement ties (in tension). They emphasized critical design parameters such as strut angles (typically 45°–55°), effective depth, and horizontal projection. Their model was validated through extensive scaled and full-scale testing at CEREBTP and led to practical guidelines, including reinforcement area calculations and a recommended safety factor of 1.15 for two-pile caps. Building upon their work, the study introduces inclined bars arranged in a strut-and-tie configuration as novel reinforcement. Compared to conventional mesh reinforcement, this layout significantly enhanced ultimate load capacity and delayed structural failure, as confirmed experimentally. Clarke^13^ investigated the structural behavior of RC pile caps supported by four piles by testing fifteen half-scale specimens with varying pile spacing, reinforcement arrangements, and anchorage details. Most specimens failed in shear following the yielding of longitudinal reinforcement, revealing the inadequacy of traditional sectional shear design. The study endorsed the truss analogy as a more accurate method for analyzing load transfer in pile caps. Additionally, bunched square reinforcement increased load capacity by approximately 25% over grid patterns but reduced crack control. The findings advocate for strut-and-tie modeling and improved reinforcement detailing in pile cap design. Adebar et al.^14^ conducted experiments on completed scaled pile beams designed using both sectional and STM approaches. Results showed that STM more accurately predicted load capacity, while the sectional approach was inadequate due to its reliance on effective depth. They concluded that pile caps experienced premature failure from longitudinal splitting of concrete struts and recommended limiting the bearing stress to 1.0 $\:{f}_{c}^{{\prime\:}}$ (compressive strength of concrete). Sam and Iyer^15^ investigated rectangular four-pile cap specimens to examine reinforcement arrangement effects. Pile caps with uniformly distributed reinforcement demonstrated superior load capacity and stiffness compared to those with bunched reinforcement, highlighting the impact of reinforcement distribution on structural performance. Ateş and Şadoğlu^16^ investigates the load sharing ratio (LSR) between piles and raft in PRFs using tests and ABAQUS simulations. Results show rafts significantly contribute to load transfer. Pile length impacts LSR by 11–14%, while relative density has a minor effect (1–2%). Settlements decrease with PRFs. Gu et al.^17^ conducted experiments on four one-fifth scale four-pile cap samples to study effect of tension reinforcing arrangements on ultimate load and collapse modes. All samples collapsed in corner-pile punching and shear regardless of reinforcement distribution. Diagonal steel bars improved load capacity, whilst square reinforcing enhanced both strength and ductility, demonstrating its superior performance. Souza et al.^18^ developed an STM capable of predicting the cracking load, steel yielding, failure behavior, and peak capacity of samples. So, the model excluded the influence of concrete’s tensile strength.

Researchers have increasingly utilized finite element modeling as a modern approach to analyze the impact of critical parameters affecting the behavior of RC pile caps. To study the connection type between RC pile caps and supporting steel piles, Gonçalves et al.^19^ developed a three-dimensional nonlinear finite element model using the ANSYS software. Additionally, the model was further utilized to analyze how pile embedment length, concrete compressive strength, and welded bars on steel piles—both with and without stirrups—affected the failure load and failure mode of RC pile caps. Delalibera and Giongo^20^ numerically determined that increasing the pile embedment length within the cap significantly improved the bearing capacity of the RC pile cap-to-pile connection. Additionally, Iyer and Sam^21^ proposed a three-dimensional elastic analysis method, treating concrete as a linearly elastic, homogeneous, and isotropic material. While offering a preliminary solution, the method lacked extensive research specific to samples. Sam and Iyer^15^ introduced a FE modelling to analyze 4 samples, addressing elastic analysis limitations. However, it underestimated behavior of the cracks for samples.

Despite extensive research on reinforced concrete (RC) pile caps, the effects of column and pile geometry and configuration on shear performance remain insufficiently explored. This study addresses this gap by experimentally and numerically investigating the structural behavior of RC pile caps, focusing on how variations in column and pile dimensions influence shear capacity. Two pile cap specimens with different heights were tested, and a validated 3D finite element model was developed using ABAQUS v.2021^22^ to simulate various rectangular and circular column–pile configurations, with dimension ratios ranging from 0.2 d to d (where d is the pile cap width). The study also examines failure modes, load–displacement responses, peak loads, and energy absorption capacities. These findings enhance the understanding of RC pile cap performance and support the development of more efficient and reliable foundation designs.

## Experimental program

The experimental study involves two reinforced concrete (RC) pile caps of identical dimensions but varying thicknesses. The upcoming sections will provide an in-depth explanation of the methodology, including the specifics of the specimens, material characteristics, preparation process, and the testing setup and loading configuration.

### Materials

#### Normal concrete

**Fig. 1:**
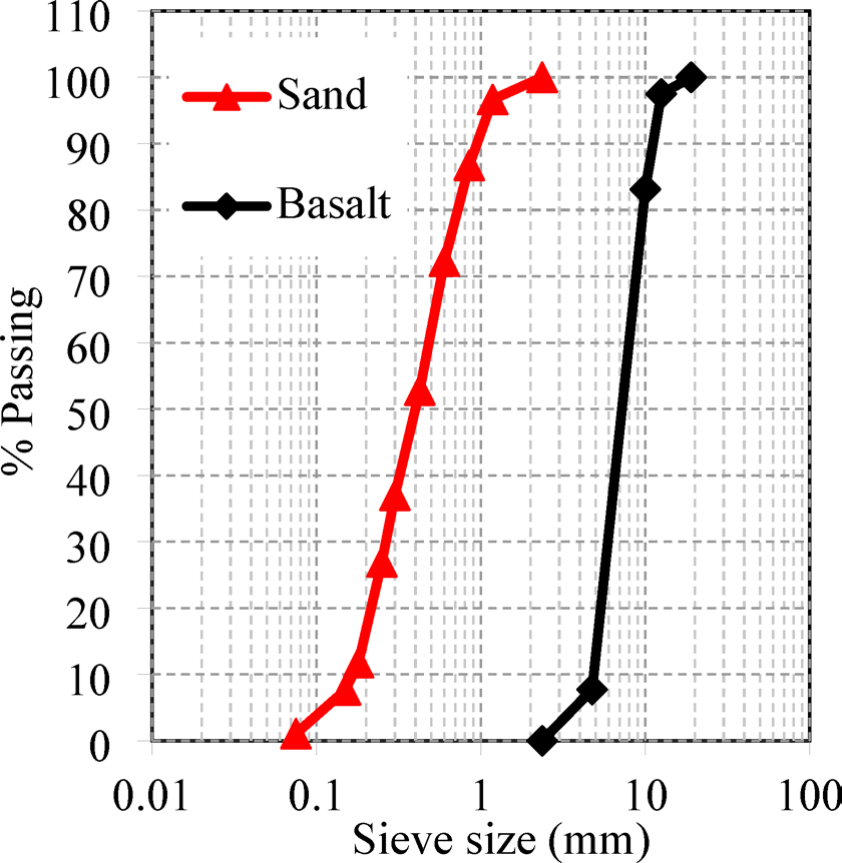
Distribution of materials utilized in the mix.

The two tested pile caps were cast using normal concrete (NC). As detailed in Table 1, the NC mix comprised sand as the fine aggregate, crushed basalt dolomite (with a maximum particle size of 15 mm) as the coarse aggregate, Portland cement (Grade 42.5), potable water, and a superplasticizer. The concrete mix was proportioned using a ratio of 1:2.17:4.3 (cement: fine aggregate: coarse aggregate) with a water-to-cement (w/c) ratio of 0.5. To improve the workability of the fresh concrete, Sikament-163 M was added as a superplasticizer. The mechanical sieve analysis results for both sand and crushed basalt are shown in Fig. 1, while Table 2 summarizes the physical properties of the NC mix components. To determine the mechanical properties of the concrete, compressive strength tests were carried out in accordance with the Egyptian Code of Practice (ECP 203/2018)^23^. Three standard cylindrical specimens (150 × 300 mm) were cast and cured under the same conditions as the test specimens, and tested at 28 days after casting. The average compressive strength obtained from the three cylinders was 30 N/mm². These results confirm the suitability of the mix for structural applications and provide a reliable basis for numerical modeling.

**Table 1:** Compositions of utilized concrete (kg/m^3^).

Concrete mix type	Cement	Water	Fine aggregate	Coarse aggregate	Super plasticizer	water/cement (%)
NC	300	150	655	1295	2.5	0.50

**Table 2:** Features of materials used.

Material	Ultimate dimension of particles		Fineness modulus	Weight (kg/m^3^)	Specific weight
	Diameter (mm)	Percentage (%)			
Basalt	12.5	2.51	2.11	1233.7	2.43
Sand	1.18	3.34	6.07	1302	2.67
Cement	0.075	13.8	No	1206	3.13

#### Reinforcing rebar

**Fig. 2:**
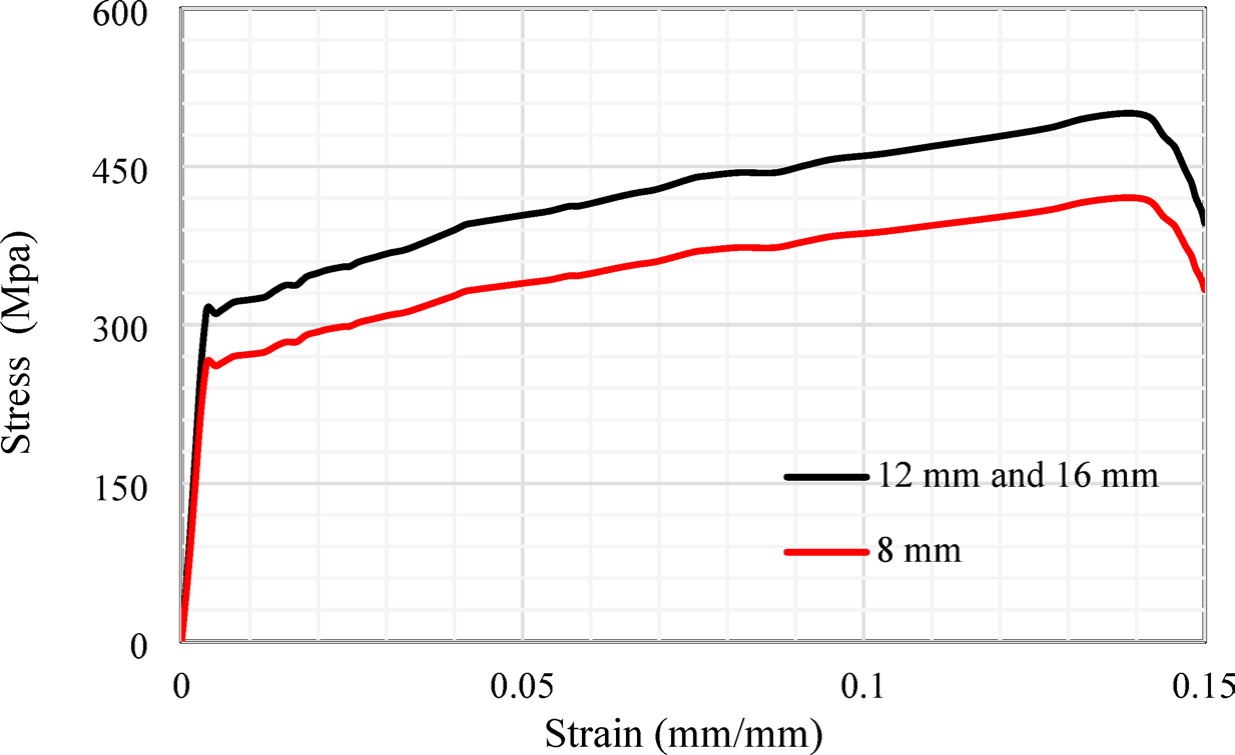
Stress-strain curves of the reinforcing bars.

The experimental study employed two types of steel rebars: normal mild steel (NMS) and high tensile steel (HTS). The NMS rebars featured a smooth surface with an 8 mm diameter, while the HTS rebars, characterized by ribbed surfaces, were available in diameters of 12 mm and 16 mm. 8 mm steel bars were incorporated as transverse reinforcing. The remaining steel bars were designated for use as main reinforcement, secondary reinforcement, and side reinforcement. Tensile tests were conducted in compliance with ECP^23^ to assess the mechanical characteristics of the steel bars used. Figure 2 illustrated the tension stress versus elongation of the rebars. The Ø12 mm and Ø16 mm reinforcement bar showed yield and ultimate tensile strengths of 317 MPa and 500 MPa, respectively. The Ø8 mm bars exhibited yield and ultimate tensile strengths of 266 MPa and 420 MPa, as outlined in Table 3.

**Table 3:** Rebars used.

Type	Bar	Surface condition	Use	Poisson’s ratio	Elastic modulus (GPa)	Yield stress (MPa)	Ultimate stress (MPa)
NMS	8 mm	Smooth	stirrups	0.3	200	266	420
HTS	12 mm and 16 mm	Deformed	vertical	0.3	200	317	500

#### Specimen’s details

Two pile caps, labeled SB1 and SB2, were constructed using normal concrete (NC). Both pile caps measured 700 mm in length (L) and 300 mm in width (d). However, the height (h) differed, with SB1 having a height of 250 mm and SB2 a height of 350 mm. Pile caps have the same characteristics as deep beams. When the shear span-to-depth ratio of a RC beam was less than 2, this beam is considered as a deep beam, and they are usually simply supported at their ends^24–28^. Also, the American code^29^ defines deep beams as beams that are subjected to force at one side and rested at the opposite side, which enables the development of compression struts between the loads and supports. These beams satisfy one of the following conditions: (i) a clear span that is no greater than four times the height of the beam, or (ii) areas with concentrated loads located within twice the depth of the beam from the support face. In^30^, a deep beam is defined as one that has a ratio of effective span to total depth of 2.

**Fig. 3:**
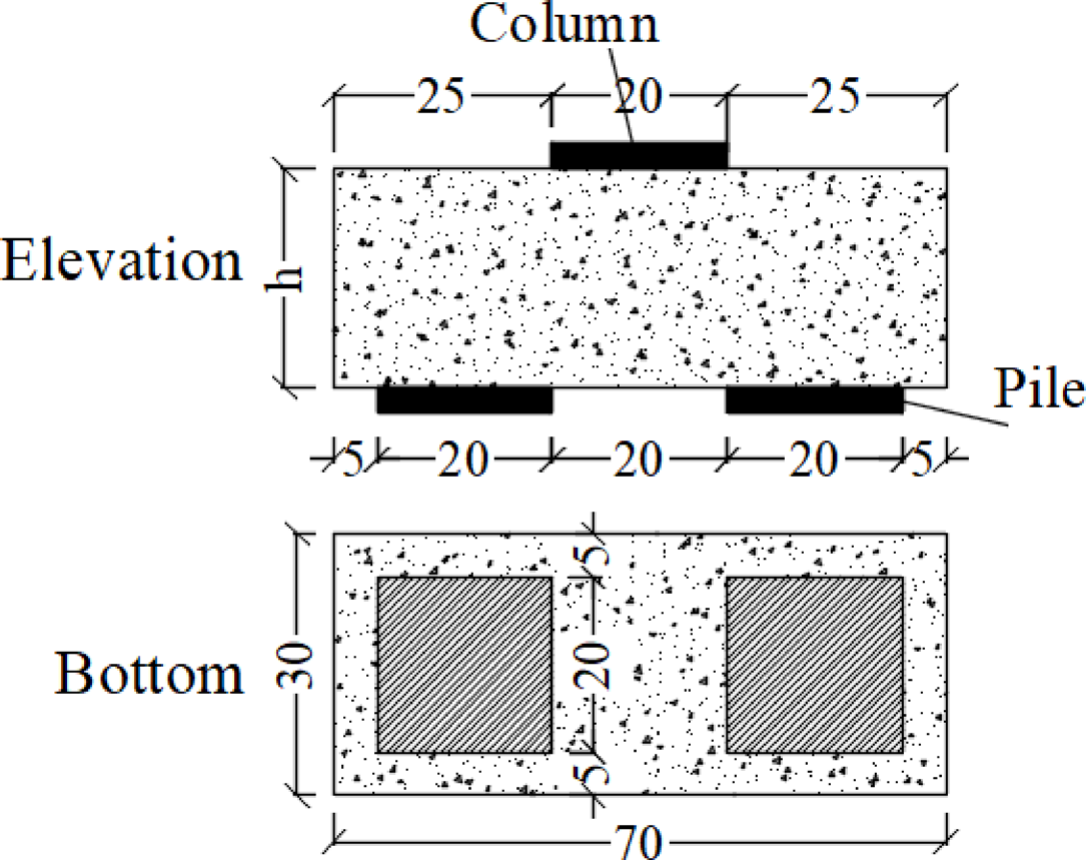
Details of pile cap beam (cm).

In the experiment, metal plates of identical size replaced the column and two piles, as depicted in Fig. 3. The dimensions of the column and piles are 200 × 200 mm. The pile caps have an effective span of 400 mm, a clear span of 200 mm, and a shear span of 250 mm for pile cap SB1 and 350 mm for pile cap SB2, all of which comply with the acceptable standards for simply supported deep beams.

**Fig. 4:**
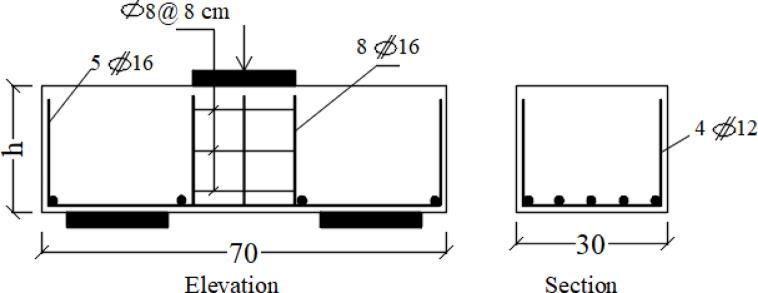
Pile cap reinforcing (cm).

**Fig. 5:**
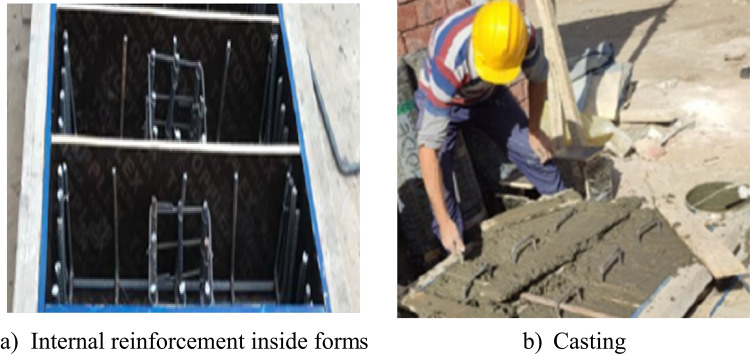
Fabrication of the tested pile caps.

Figure 4 illustrates the specifics of the internal reinforcing steel. Both pile caps share identical bottom reinforcement, which includes primary longitudinal tensile bars and secondary lateral reinforcement. The main reinforcing is made up of five HTS rebars, each with a diameter of 16 mm (5 Φ 16), while the transverse reinforcing is composed of four HTS rebars with a diameter of 12 mm (4 Φ 12). The reinforcing cage of the column consists of 8 HTS bars, each with a 16 mm diameter, and is tied together with 8 NMS bars spaced 80 mm apart. In the testing procedure, the reinforcement detailing of the pile sections embedded within the cap was intentionally simplified and did not replicate the full reinforcement typically used in actual pile construction. As shown in Fig. 4, while the reinforcement beneath the column was fully detailed, the reinforcement in the upper portion of the piles (within the cap) was limited to surrounding bars and did not fully enclose the cross-section. This approach was adopted to isolate the behavior of the cap–pile interaction, focusing on the load transfer mechanisms between the cap and the piles rather than the structural performance of the piles themselves, as also reported in previous studies^31^. Figure 5 illustrates the manufacturing process for the two pile caps that were tested.

#### Test device

**Fig. 6:**
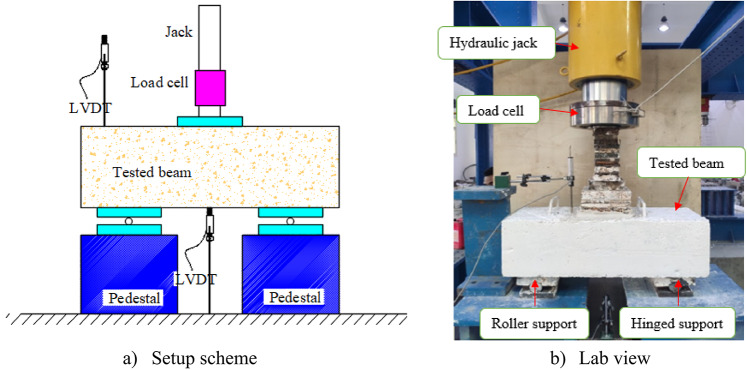
Test setup of the beam.

The experiments were carried out in a laboratory tailored for concrete structures, using a 2500 kN capacity testing machine. The 2500 kN capacity universal testing machine used in this study is a servo-controlled hydraulic system designed for high-load structural testing. It features a rigid four-column load frame and is capable of applying loads up to 2500 kN with high precision. The machine operates under displacement control, with a programmable loading rate ranging from 0.01 mm/min to 100 mm/min. It is equipped with a digital control unit and a high-accuracy load cell, ensuring load measurements within ± 1% accuracy. Before testing, the pile caps were thoroughly cleaned to eliminate dust and then coated with white paint to improve the visibility of cracks during the experiments. The steel crane was employed to lift the tested beams because of their heavy weight. Each beam was precisely positioned and held in place with two supports for the testing procedure. Figure 6 shows the layout of the testing arrangement for the pile cap undergoing the test. A Linear Variable Differential Transformer (LVDT) with a resolution of ± 0.01 mm was used to record vertical displacements, while a computerized data acquisition system provided real-time monitoring and recording of load and deflection throughout the test, as shown in Fig. 6a and b. The pile caps were incrementally loaded in 0.1 mm/min steps until they failed. Visible cracks were recorded, and deflections were measured with a data logger at each load increment. The LVDT was disconnected immediately after the failure.

## Results of tests and discussion

This section details the findings from the beam tests, focusing on critical factors such as failure modes, load-displacement behavior, ultimate shear strength, absorbed energy capacity, and ductility index.

### Failure modes

**Fig. 7:**
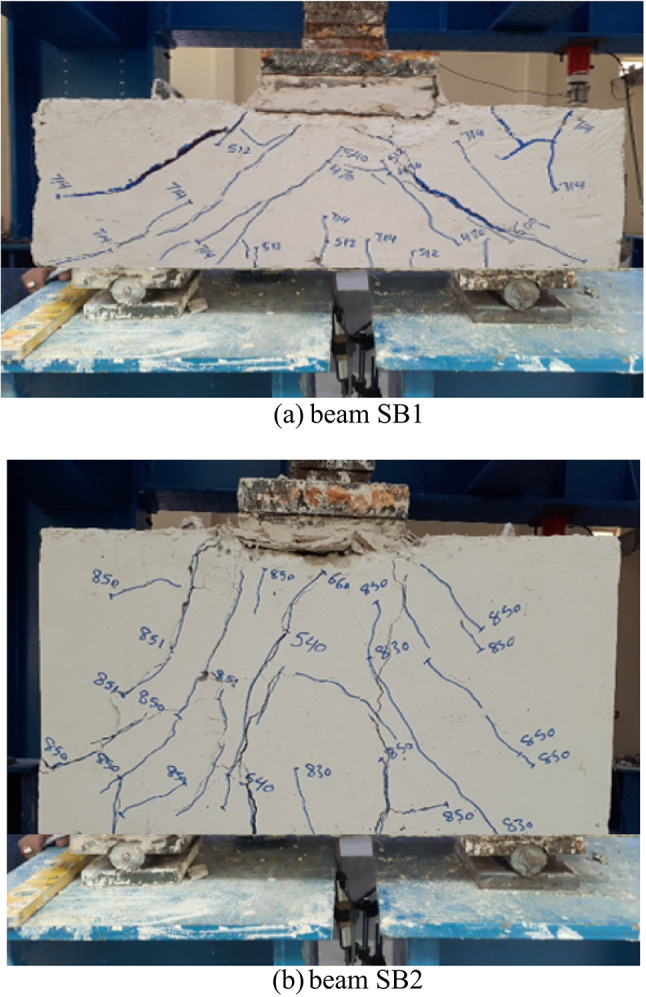
Cracks and failure modes of tested pile caps.

Cracks and failure modes were recorded at each load increment as the pile caps were tested to failure. Figure 7 shows the cracking and failure patterns observed during the pile cap tests. In general, failure in the pile caps typically occurred via shear-compression, combined with punching shear beneath the column on the top surface. Under three-point loading, deep beams with a shear span-to-depth ratio (*a/h*) of 1 failed in shear, governed by compressive stresses along 45° diagonal stress. This failure mode manifested as pure diagonal shear in pile caps SB1 and SB2. In all tested pile caps, flexural cracks initially developed. As the load increased, the large diagonal tension crack emerged near the midspan of the shear span. With further loading, additional inclined cracks propagated within the shear span, culminating in ultimate failure due to concrete crushing along the inclined crack paths. Figure 7a illustrates the crack progression in pile cap SB1. An initial diagonal shear crack formed near the support (directed toward the column) at approximately 470 kN. As loading increased, shear failure occurred at 718 kN. Similarly, Fig. 7b shows the crack development in beam SB2, where an initial diagonal shear crack appeared near the support (oriented toward the column) at 540 kN. With further loading, shear failure ensued at 851 kN. These findings align with prior experimental studies^12,13^, where shear failures were predominant in similarly proportioned pile caps.

### General performance

**Fig. 8:**
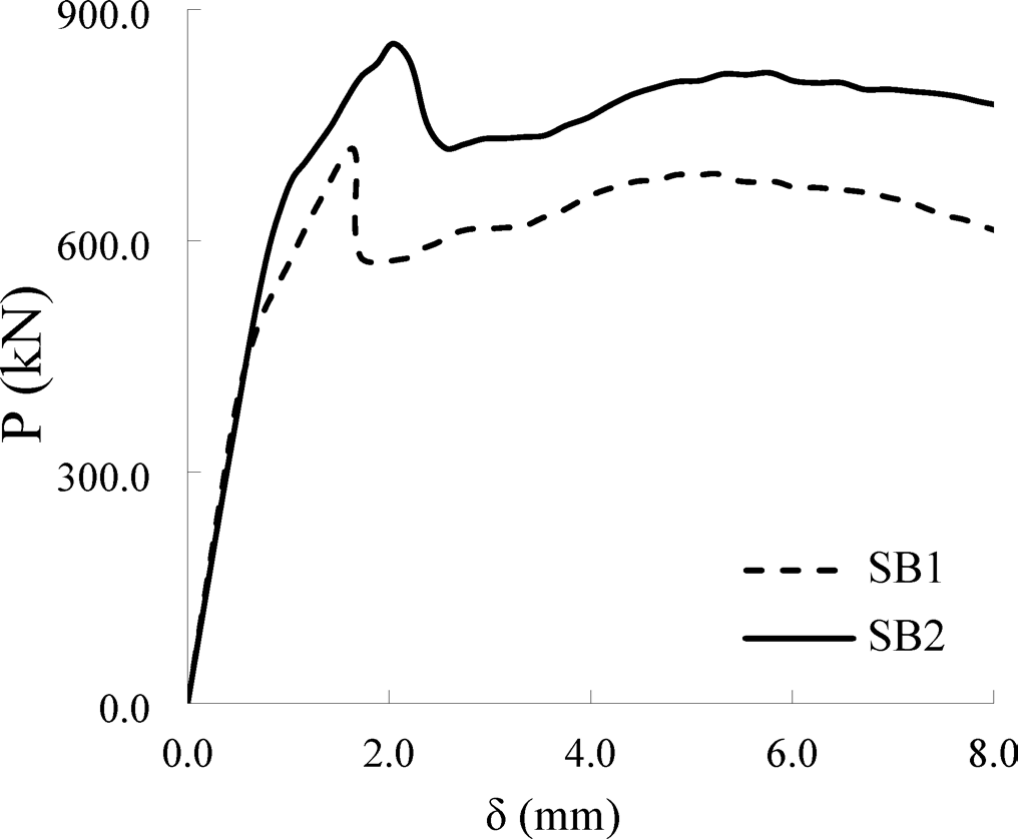
Load- deflection curves of tested pile caps.

The applied force versus displacement (*P-δ*) curves of the test pile caps at various loading stages are shown in Fig. 8. Meanwhile, the ultimate load, shear ductility index (*λ*), and energy dissipation capacity I of the tested pile caps—which characterize their ductility properties—are summarized in Table 4.

The load-deflection (*P- δ*) curves generally exhibit four distinct stages (Fig. 8). Stage I (linear elastic phase) spans from initial loading to approximately 60–80% of the peak load. During this phase, the load (*P*) increases rapidly, while mid-span deflection (*δ*) progresses slowly. Stage II (nonlinear pre-peak phase) begins at the end of Stage I and extends to the peak load. Here, the deflection rate accelerates significantly due to the initiation of the first crack, while the load increases only marginally. Cracking intensifies as the curve approaches the peak, followed by a post-peak load drop. Stage III (rapid post-peak decline) is marked by a sharp nonlinear reduction in load as cracks propagate and widen, causing abrupt structural softening. Finally, Stage IV (residual phase) stabilizes into a near-horizontal trend, where deflection continues to increase under minimal load resistance. The four-stage response mirrors the nonlinear phases reported by Sam and Iyer^15^ and Souza et al.^18^, particularly the post-peak softening attributed to shear crack widening.

In addition, the variables exhibited no discernible impact on the *P-δ* relationship during the initial linear elastic phase across all tested samples. However, their influence became pronounced in subsequent nonlinear stages. The results showed that increasing the beam height by 40% (35 cm) led to a noticeable improvement in the beams’ behavior regarding the load-deflection relationship (the beam’s ability to withstand loads while deforming). Pile cap SB2 recorded the highest shear resistance **(**maximum shear strength**)** and the largest deflection before failure, indicating enhanced durability (energy absorption capacity) and ductility (ability to deform without sudden collapse). The enhancement in ultimate load and energy absorption with increased column/pile dimensions is supported by Delalibera and Giongo’s numerical studies^20^, which highlighted the role of bearing area in load transfer.

**Table 4:** Summary of the test pile caps results.

Height	Parameter	Specimen	Ultimate load		Energy absorption		Ductility Index	
			*P*_*u*_ (kN)	*P*_*u*_ */P*_*u, o*_	*E* (kN.mm)	*E/E*_*o*_	_*λ*_	$\lambda/\lambda_o$
250	Pile Cap height	SB1	718	1.00	5616.4	1.00	2.28	1.00
350		SB2	851	1.18	7746.5	1.40	2.80	1.23

$\:{P}_{u}$: Ultimate load of the tested pile caps,$\:{P}_{uo}$: Ultimate load of specimen SB1, λ : Ductility index of the tested pile caps,$\:{\lambda\:}_{o}$: Ductility index of specimen SB1. .

Specimens SB1 and SB2 achieved ultimate loads ($\:{P}_{u}$) of 781 kN and 851 kN, accompanied by mid-span deflections of 1.65 mm and 2.04 mm, respectively. The increased height of SB2 improved its structural stiffness, culminating in a 18% higher ultimate load and 24% greater ultimate deflection compared to SB1.

**Fig. 9:**
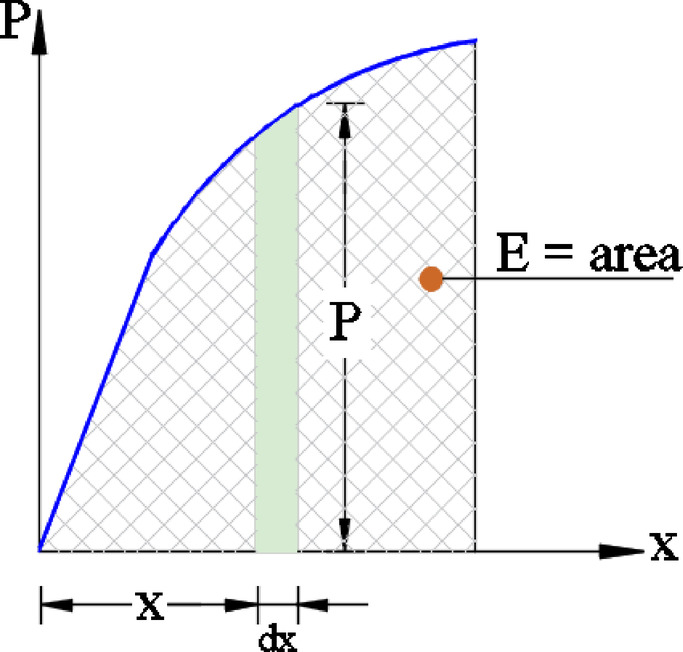
Absorbed energy assessment.

Additionally, the absorbed energy (E) for the tested pile caps is determined by calculating the area under the measured load-deflection curves^32–34^. As depicted in Fig. 9, the total absorbed energy (E) is derived by integrating the force (P) with respect to displacement ($\:{d}_{x}$) across the range $\:x=0$ to $\:x={x}_{1}$, expressed by:

1 $$\:E={\int\:}_{0}^{{X}_{1}}P\:dx$$

The ductility index (λ), on the other hand, is defined as the ratio of the ultimate deflection to the yield deflection. The calculated absorbed energy and ductility index values for the tested pile caps are presented in Table 4. The table indicates that pile cap SB2 exhibits a 40% increase in absorbed energy (7746.5 kN·mm) and a 23% increase in ductility index (2.80) compared to beam SB1, which has an absorbed energy of 5616.4 kN·mm and a ductility index of 2.28.

## Numerical simulation

The experimental records were compared to a three-dimensional (3D) non-linear finite element model (FEM) that was built using ABAQUS v.2021^22^. To accurately predict the shear performance of RC pile caps, a numerical analysis was performed. The ABAQUS v.2021 was selected for the numerical analyses due to its advanced capabilities in simulating complex non-linear behavior, contact interactions, and load applications, as well as its ability to provide accurate predictions of the structural performance of RC pile caps. Its robust features for modeling material non-linearity and handling large deformations make it an ideal choice for this type of analysis^35–39^.

### Finite element modeling

**Fig. 10:**
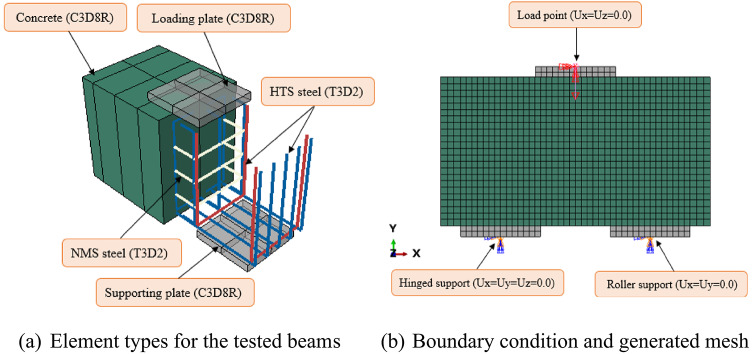
FEM of the tested pile caps.

The proposed model was developed to simulate concrete and steel plates utilizing eight-point linear brick solid components (C3D8R). Each element incorporates three translational degrees of freedom, as depicted in Fig. 10. In addition, 2-node linear 3D truss elements (T3D2), characterized by axial stiffness only, were utilized to simulate reinforcement.

**Fig. 11:**
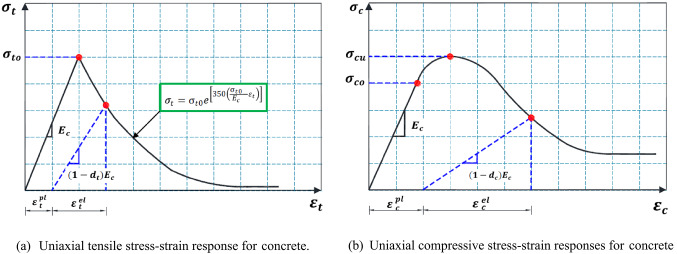
Uniaxial compressive and tensile stress–strain responses^50^.

Normal concrete (NC) was modeled using the Concrete Damage Plasticity Model (CDPM) to evaluate its softening behavior and strain-hardening characteristics^40–49^. To develop the CDPM, two essential properties of concrete were required: compressive crushing and tensile cracking^50^. Figure 11(a) illustrates the concrete’s tensile uniaxial response. The tensile stress-strain behavior is linear up to the failure stress, $\:{\sigma\:}_{to}$. Once the tensile stress-strain curve begins to soften, micro-cracks develop beyond the failure stress. Additionally, Fig. 11(b) illustrates the compressive stress–strain response. The response was linear up to the first yield stress, $\:{\sigma\:}_{co}$. Beyond this point, the stress-strain curve exhibited a hardening trend, ultimately reaching the compressive ultimate stress, $\:{\sigma\:}_{CU}$. The compressive stress-strain curve demonstrated stress softening behavior after exceeding the compressive ultimate stress. ABAQUS has the capability to automatically transform user-defined uniaxial stress-inelastic strain curves into stress versus plastic-strain curves, as shown in Eqs. (2) and (3).

2 $$\:{\sigma\:}_{t}={\sigma\:}_{t}({\varepsilon\:}_{t}^{pl},\:{\varepsilon\:}_{t}^{{\prime\:}\:pl})$$

3 $$\:{\sigma\:}_{c}={\sigma\:}_{c}({\varepsilon\:}_{c}^{pl},\:{\varepsilon\:}_{c}^{{\prime\:}\:pl})$$

where tension and compression are indicated by the subscripts t and c, respectively. The tensile and compressive stresses of the concrete are denoted by $\:{\sigma\:}_{t}$ and $\:{\sigma\:}_{c}$, respectively. $\:{\varepsilon\:}_{t}^{pl}$ and $\:{\varepsilon\:}_{c}^{pl}$represent the equivalent plastic strains. While $\:{\varepsilon\:}_{t}^{{\prime\:}pl}$ and $\:{\varepsilon\:}_{c}^{{\prime\:}pl}$ represent the equivalent plastic strain rates.

The compressive and tensile damage parameter formulations proposed by Birtel and Mark^51^ were utilized to model the failure processes of concrete material under tension and compression loadings, as presented in Eqs. (4) and (5).

4 $$\:{d}_{t}=1-\frac{{\sigma\:}_{t}{E}_{c}^{-1}}{{\varepsilon\:}_{t}^{pl}(1/{b}_{t}-1)+{\sigma\:}_{t}{E}_{c}^{-1}}$$

5 $$\:{d}_{c}=1-\frac{{\sigma}_{c}{E}_{c}^{-1}}{{\varepsilon\:}_{c}^{pl}(1/{b}_{c}-1)+{\sigma}_{c}{E}_{c}^{-1}}$$

where the tensile and compressive damage parameters are denoted by $\:{d}_{t}$ and $\:{d}_{c}$, respectively. The constant parameters, $\:{b}_{t}$ and $\:{b}_{c}$, range between 0 and 1.

The Saenz model^52^ was utilized to provide a detailed representation of concrete behavior under uniaxial compression, as outlined in Eqs. (6)–(12)^53^.

6 $$\:{\sigma\:}_{c}=\frac{{E}_{c}{\varepsilon\:}_{c}}{1+\left(R+{R}_{E}-2\right)\frac{{\varepsilon\:}_{c}}{{\varepsilon\:}_{o}}-\left(2R-1\right){\left(\frac{{\varepsilon\:}_{c}}{{\varepsilon\:}_{o}}\right)}^{2}+R{\left(\frac{{\varepsilon\:}_{c}}{{\varepsilon\:}_{o}}\right)}^{3}}$$

7 $$\:{E}_{c}=4700\sqrt{{f}_{c}^{{\prime\:}}}\:\:\:\:\:\:\text{for normal strength concrete}$$

8 $$\:R=\:\frac{{R}_{E}({R}_{\sigma\:}-1)}{{\left({R}_{\varepsilon\:}-1\right)}^{2}}-\frac{1}{{R}_{\varepsilon\:}}$$

9 $$\:{R}_{E}=\frac{{E}_{C}}{{E}_{O}}$$

10 $$\:{R}_{\sigma\:}=\frac{{f}_{c}^{{\prime\:}}}{{\sigma\:}_{f}}$$

11 $$\:{R}_{\varepsilon\:}=\frac{{\varepsilon}_{f}}{{\varepsilon}_{o}}$$

12 $$\:{E}_{O}=\frac{{f}_{c}^{{\prime\:}}}{{\varepsilon}_{O}}$$

Where $\:{\sigma}_{c}$ was the compression strength of the concrete, $\:{E}_{c}$ was concrete elasticity index, $\:{E}_{O}$ was concrete secant elasticity, $\:{f}_{c}^{{\prime\:}}$ was ultimate compression, $\:{\varepsilon}_{c}$ was the strain value in the compression; $\:{\varepsilon\:}_{o}$ was corresponding strain at the $\:{f}_{c}^{{\prime\:}}$ value (equals about 0.0025^54^), $\:{\varepsilon}_{f}$ was peak strain, $\:{\sigma}_{f}$ was pressure related to $\:{\sigma}_{f}$, $\:{R}_{E}$ was modular factor, $\:{R}_{\sigma\:}$ was ratio of the pressure (taken 4^54^), $\:{R}_{\varepsilon\:}$ was ratio of strain (taken 4^54^).

The uniaxial tensile stress–strain behavior of concrete was characterized by two phases: an initial linear elastic response and a subsequent softening curve. For an initial linear elastic response, the elastic modulus ($\:{E}_{C}$) and tensile strength ($\:{\sigma\:}_{to}$) were critical for characterizing the linear response of each material. Specifically, for concrete, the elastic modulus $\:{E}_{C}$ was defined according to Eq. 8. In addition, as illustrated in Fig. 11a, the strain-hardening behavior was modeled to match the experimental observations (Fig. 2), while the tensile softening branch was calculated using Eq. 13, following the methodology outlined by Ganganagoudar et al.^55^. For concrete, the Poisson’s ratio was assigned a value of 0.2, while the plasticity parameters for the CDPM were defined as provided in Table 5^24–28,33^.

13 $$\:{\sigma\:}_{t}={\sigma\:}_{to}{e}^{\left[350\left(\frac{{\sigma\:}_{to}}{{E}_{c}}\:-\:{\varepsilon\:}_{t}\right)\right]}$$

Where$\:\:{\varepsilon\:}_{t}$ denotes the tensile strain associated with $\:{\sigma\:}_{t}$​, the tensile strength.

**Table 5:** Parameters used of CDP model.

η	$\:{f}_{bo}/{f}_{co}$	$\:{K}_{c}$	ψ	e
0.00	1.16	0.667	35°	0.10

µ: viscosity parameter, $\:{f}_{bo}/{f}_{co}$: ratio of biaxial to uniaxial compressive yield stresses, $\:{K}_{c}$ : ratio of the second stress invariant on the tensile to the compressive meridian, ψ: dilation angle, and e: eccentricity.

Conversely, a perfectly plastic elastic response, exhibiting identical properties in tension and compression, was adopted to model the steel reinforcement’s behavior in the case of shear failure. The elastic-perfectly-plastic model can be considered suitable when the steel bars’ ultimate axial loads are less than or nearly equivalent to the yield load. However, in cases of flexural failure accompanied by steel yielding, the steel reinforcement was represented using an elastic-plastic model that included strain hardening. The elastic modulus and Poisson’s ratio of the steel were set to 200 GPa and 0.3, respectively, while the yield stress was determined through experimental tests, as summarized in Table 5.

Since the steel reinforcement did not slip from the RC pile caps due to indentations and sufficient development, the interaction between the embedded reinforcing bars, stirrups, and the surrounding concrete was modeled using a fully bonded embedded region constraint^56–59^. For the analysis in this study, the static-general procedure was selected due to its enhanced robustness and reliability compared to the static-risk or dynamic-explicit approaches.

**Fig. 12:**
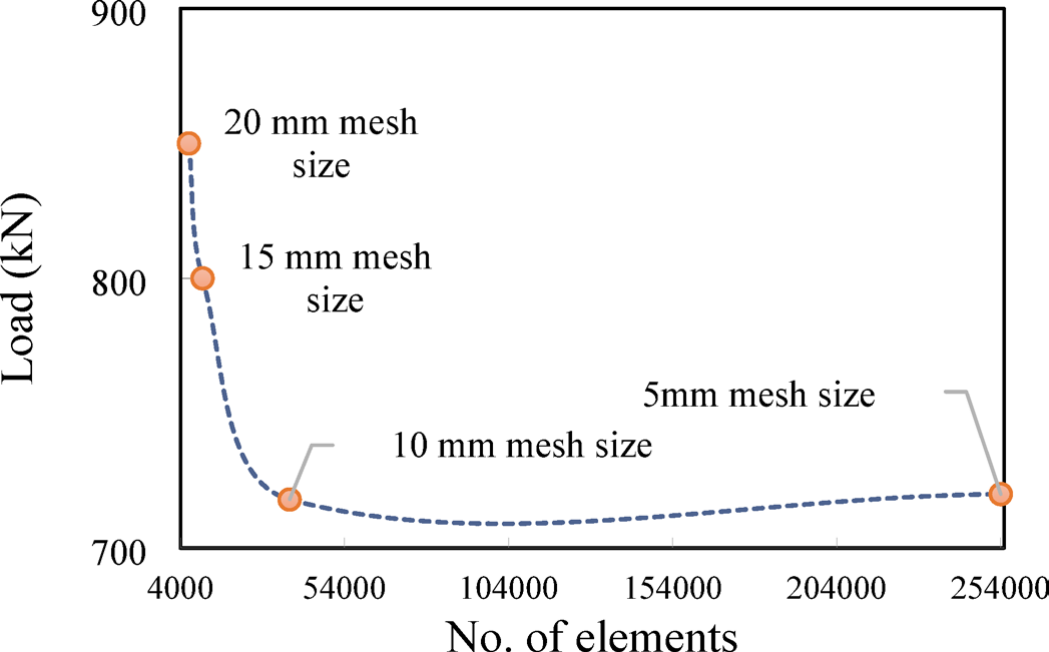
Load level for different mesh sizes.

To identify the most suitable mesh size for the tested beams, a mesh sensitivity analysis was carried out. The results indicated that a 10 mm mesh size delivered a reliable representation across all models, ensuring sufficient accuracy while minimizing computational expense. The conclusion was based on experiments utilizing mesh sizes from 5 mm to 20 mm, with results compared against experimental data, as illustrated in Fig. 12.

### Numerical outcomes

**Fig. 13:**
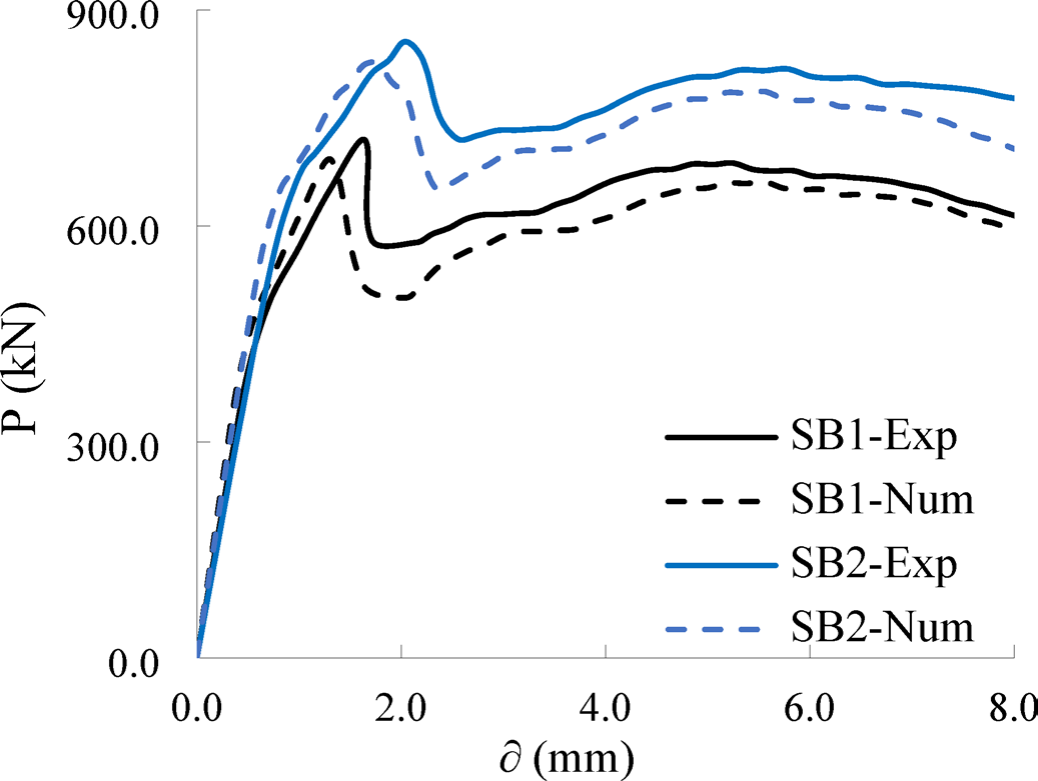
Experimental and numerical load-deflection relationships for tested pile caps.

**Fig. 14:**
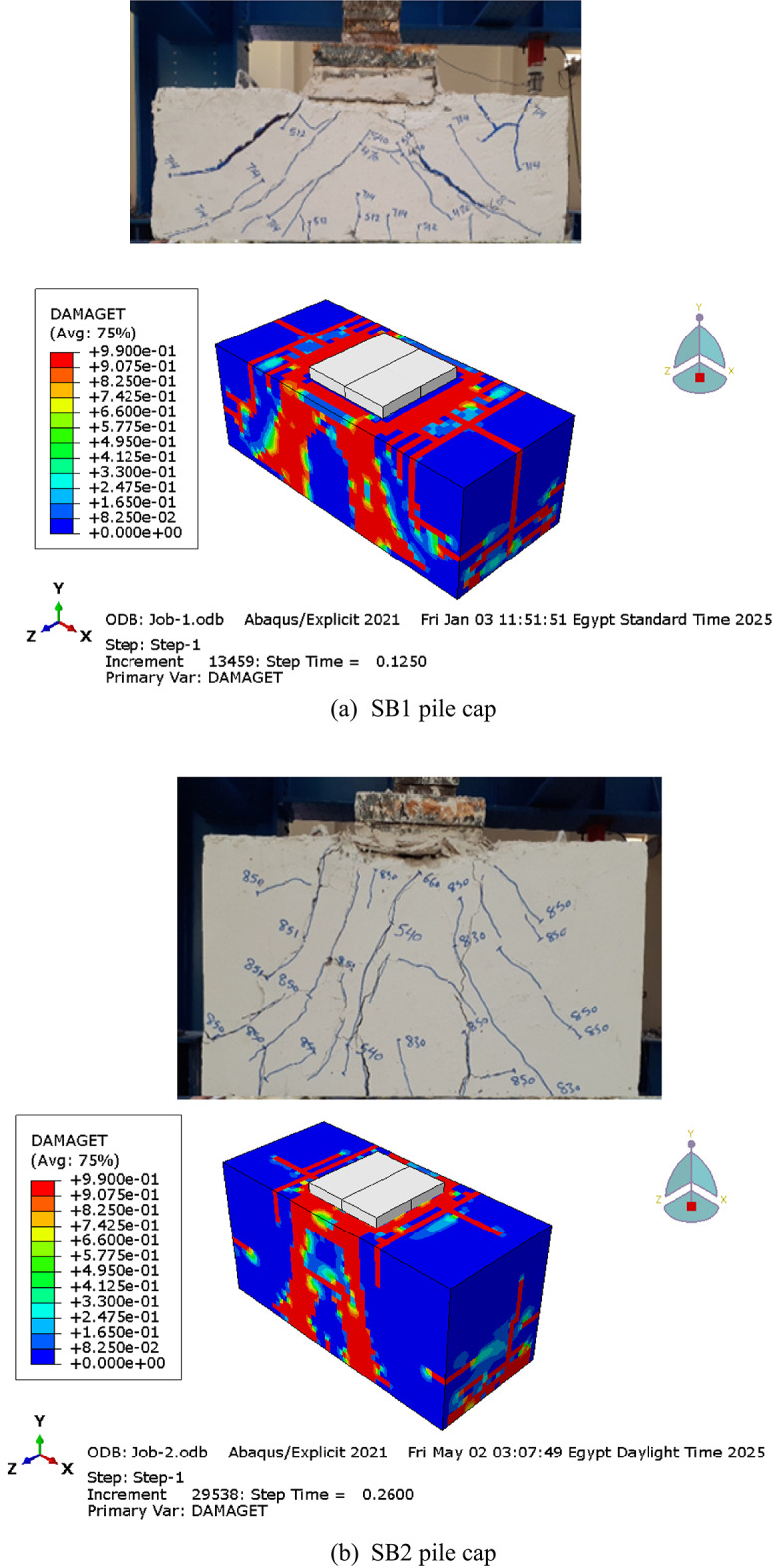
Comparison of experimental and numerical failure patterns from (tensile damage/DAMAGET).

The computational results were thoroughly validated against experimental data, with a focus on load-deflection response and crack patterns (Figs. 13 and 14). Figure 13 presents the load–deflection curve extracted from the finite element model and compared against experimental data. In the FEM analysis, the deflection was obtained from the vertical displacement of the node located at the geometric center of the column base. This point corresponds precisely to the location where displacement measurements were recorded during the experimental tests, ensuring consistency and accurate comparison between numerical and experimental results. By monitoring the displacement at this central node throughout the loading process, the simulation effectively captured the overall flexural response of the pile cap system under vertical loading. Key values extracted from the load-deflection curves are summarized in Table 5.

Figure 14 illustrates the simulated crack patterns and failure mechanisms of the tested pile caps, demonstrating the model’s ability to replicate the observed experimental behaviors. The FEM results accurately reflected the influence of pile cap height on failure mechanisms and cracking patterns, validating the model’s capability in capturing structural responses under different configurations. Furthermore, as summarized in Table 6, the reliability of the FEM model was quantitatively verified: the mean ratio of experimental to numerical ultimate loads ($\:{P}_{u,EXP}/{P}_{u,FE}$) was 0.96, with a low coefficient of variation (CoV) of 0.02 and a standard deviation (SD) of 0.02. These results confirm the high accuracy and reliability of the developed numerical model in predicting the structural behavior of RC pile caps.

**Table 6:** Results from experiments and numerical simulations for all tested pile caps.

Specimen	$\:{P}_{u,EXP}$ (MPa)	$\:{P}_{u,FE}$ (MPa)	$\:\frac{{P}_{u,EXP}}{{P}_{u,FE}}$
SB1	719	691	1.04
SB2	855	826	1.035
µ			1.0378
SD			0.0027
CoV (%)			0.0026

Where, µ : mean deviation, SD: Standard Deviations, CoV: Coefficient of Variation, $\:{P}_{u,EXP}$ : Experimental ultimate load, $\:{P}_{u,FE}$: Numerical ultimate load.

## Parametric analysis

**Fig. 15:**
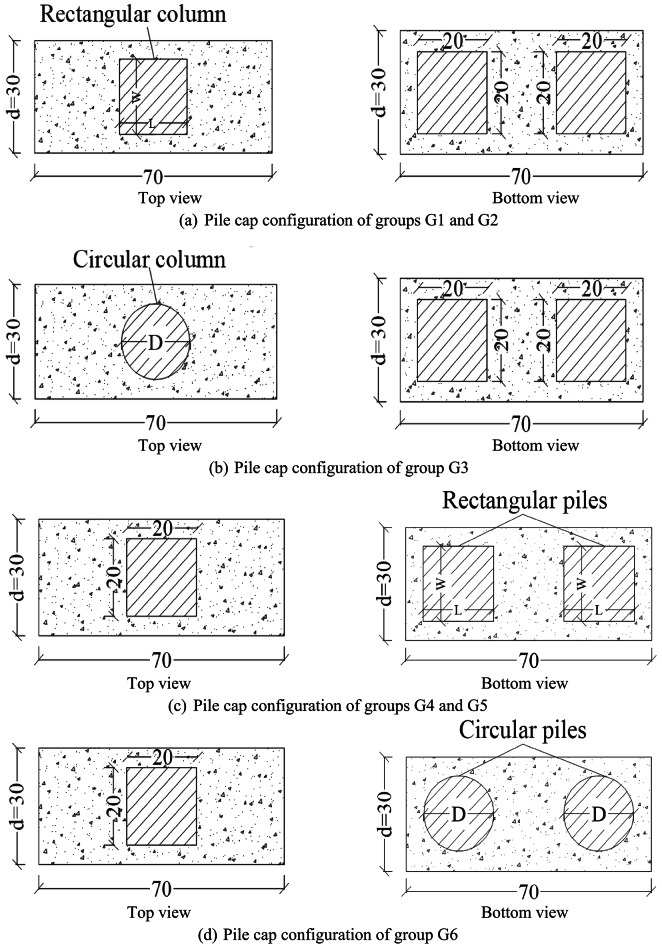
Pile cap configurations used in parametric analysis (cm).

In the current study, a parametric analysis was conducted using the validated model to investigate the effect of column and pile dimensions and their configurations on the shear behavior of RC pile caps. Thirty RC pile caps with two distinct configurations of columns and piles (rectangular and circular) were proposed, featuring varying dimensions for both, as illustrated in Fig. 15. The specimen SB2 was utilized as the control pile cap, featuring a square column and piles with dimensions of 200 × 200 mm. The thirty specimens shared the same pile cap dimensions and internal reinforcing steel configuration as SB2.

The tested pile caps were divided into six groups for the evaluation of shear performance. Each group included specimens with similar characteristics, with one variable adjusted to assess its impact on performance, as outlined in Table 7. As shown in Table 7, groups G1 and G2 investigated the effect of rectangular column configurations on the shear performance of pile caps. G1 analyzed the effect of column length on pile cap shear behavior using five specimens (SB2-CL-0.2 d to SB2-CL-d) with lengths varying from 0.2 d (60 mm) to d (300 mm). G2 examined the effect of column width with five specimens (SB2-CW-0.2 d to SB2-CW-d) having widths ranging from 0.2 d (60 mm) to d (300 mm), where d represents the pile cap width. However, group G3 examined the effect of circular column configurations on the shear performance of pile caps. This group consisted of five specimens (SB2-CD-0.2 d to SB2-CD-d) with diameters ranging from 0.2 d (60 mm) to d (300 mm).

Additionally, G4 and G5 investigated the effect of rectangular piles configurations on the shear performance of pile caps. G4 analyzed the effect of pile length on pile cap shear behavior using five specimens (SB2-PL-0.2 d to SB2-PL-d) with lengths varying from 0.2 d (60 mm) to d (300 mm). G5 examined the effect of pile width with five specimens (SB2-PW-0.2 d to SB2-PW-d) having widths ranging from 0.2 d (60 mm) to d (300 mm). However, group G6 examined the effect of circular piles configurations on the shear performance of pile caps. This group consisted of five specimens (SB2-PD-0.2 d to SB2-PD-d) with diameters ranging from 0.2 d (60 mm) to d (300 mm), as listed in Table 7.

**Table 7:** Details of the specimens used in the parametric analysis.

Group	Specimen`s ID	Column Dimension			Pile Dimension			Objective
		Rectangular		Circular	Rectangular		Circular	
		Length L (mm)	Width W(mm)	Diameter D (mm)	Length L (mm)	Width W (mm)	Diameter D (mm)	
Control	SB2	200	200	None	200	200	None	
G1	SB2-CL-0.2 d	60	200	None	200	200	None	Study the effect of column length
	SB2-CL-0.4 d	120	200	None	200	200	None	
	SB2-CL-0.6 d	180	200	None	200	200	None	
	SB2-CL-0.8 d	240	200	None	200	200	None	
	SB2-CL-d	300	200	None	200	200	None	
G2	SB2-CW-0.2 d	200	60	None	200	200	None	Study the effect of column width
	SB2-CW-0.4 d	200	120	None	200	200	None	
	SB2-CW-0.6 d	200	180	None	200	200	None	
	SB2-CW-0.8 d	200	240	None	200	200	None	
	SB2-CW-d	200	300	None	200	200	None	
G3	SB2-CD-0.2 d	None	None	60	200	200	None	Study the effect of column diameter
	SB2-CD-0.4 d	None	None	120	200	200	None	
	SB2-CD-0.6 d	None	None	180	200	200	None	
	SB2-CD-0.8 d	None	None	240	200	200	None	
	SB2-CD-d	None	None	300	200	200	None	
G4	SB2-PL-0.2 d	200	200	None	60	200	None	Study the effect of pile length
	SB2-PL-0.4 d	200	200	None	120	200	None	
	SB2-PL-0.6 d	200	200	None	180	200	None	
	SB2-PL-0.8 d	200	200	None	240	200	None	
	SB2-PL-d	200	200	None	300	200	None	
G5	SB2-PW-0.2 d	200	200	None	200	60	None	Study the effect of pile width
	SB2-PW-0.4 d	200	200	None	200	120	None	
	SB2-PW-0.6 d	200	200	None	200	180	None	
	SB2-PW-0.8 d	200	200	None	200	240	None	
	SB2-PW-d	200	200	None	200	300	None	
G6	SB2-PD-0.2 d	200	200	None	None	None	60	Study the effect of pile diameter
	SB2-PD-0.4 d	200	200	None	None	None	120	
	SB2-PD-0.6 d	200	200	None	None	None	180	
	SB2-PD-0.8 d	200	200	None	None	None	240	
	SB2-PD-d	200	200	None	None	None	300	

### Results and discussion of parametric analysis

#### Cracks and failure pattern

**Fig. 16:**
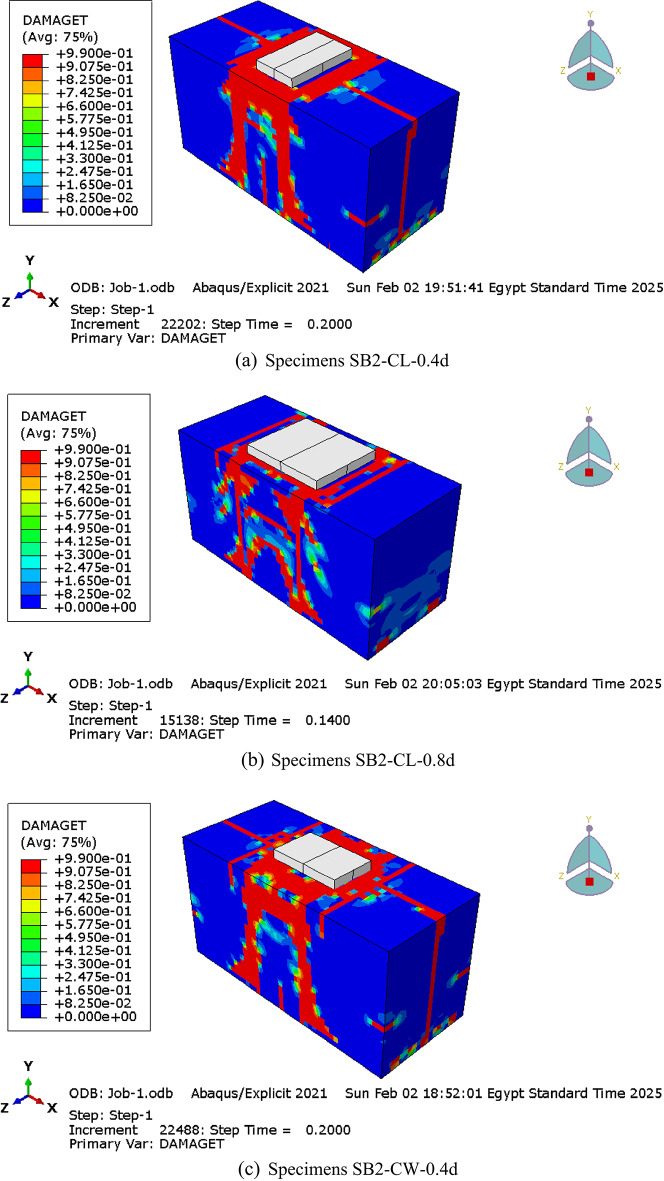

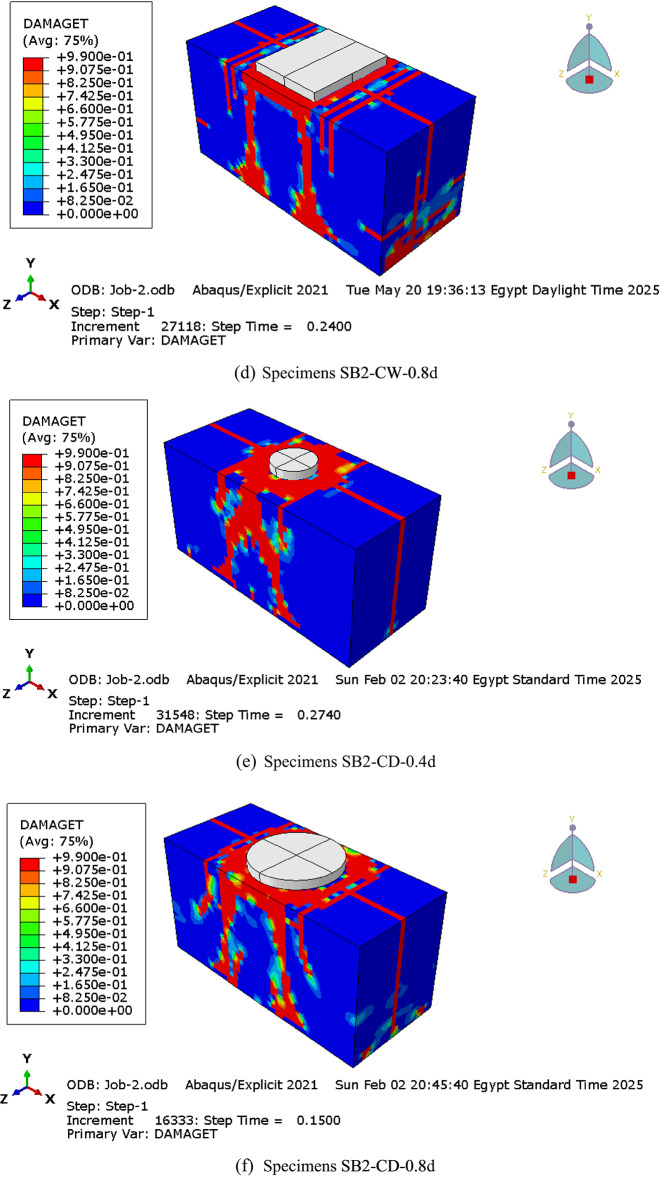
Cracks and failure patterns of parametric analysis specimens.

As previously discussed, the control pile cap (SB2) exhibited a shear failure mode due to the propagation of shear cracks within the shear zone along the diagonal direction between the support and the loading point. Regardless of the column and pile configuration or size, all RC pile cap specimens in groups G1 to G6 failed in shear, characterized by a dominant diagonal shear crack connecting the column and piles, as shown in Fig. 16. These results highlight that the column and pile size and configuration do not influence crack propagation or the failure pattern.

#### Load- deflection curves of parametric analysis specimens

**Fig. 17:**
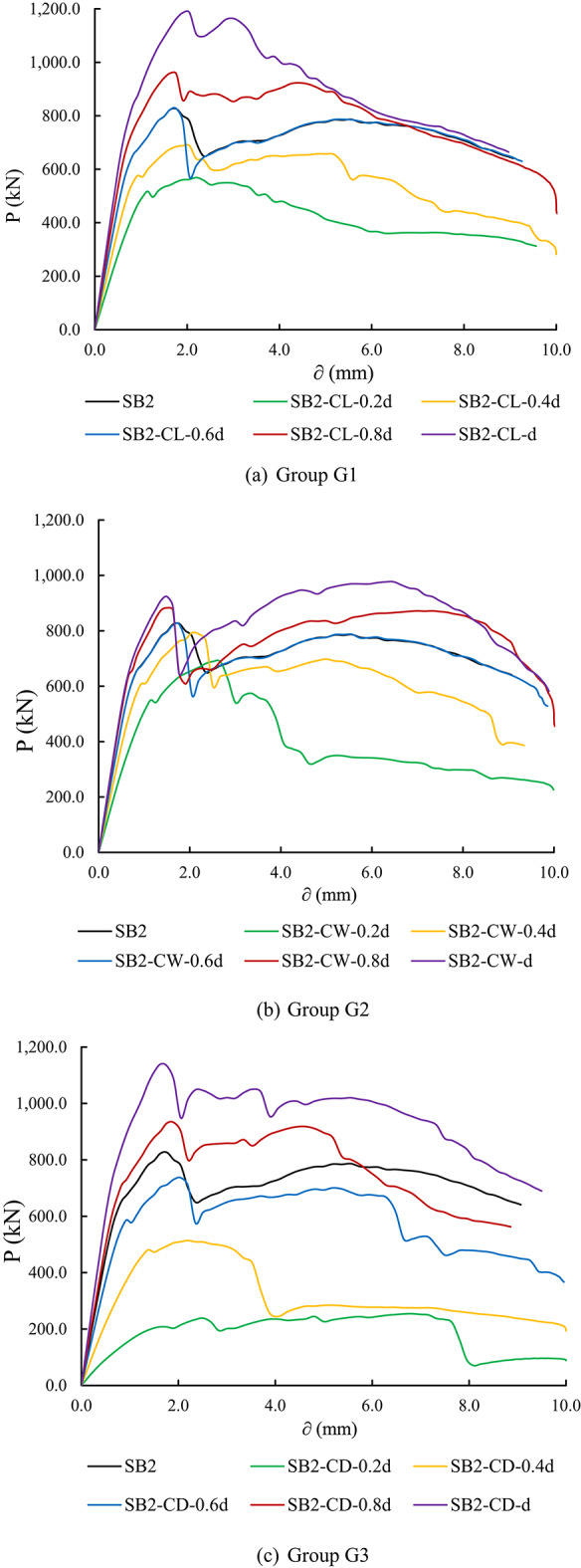

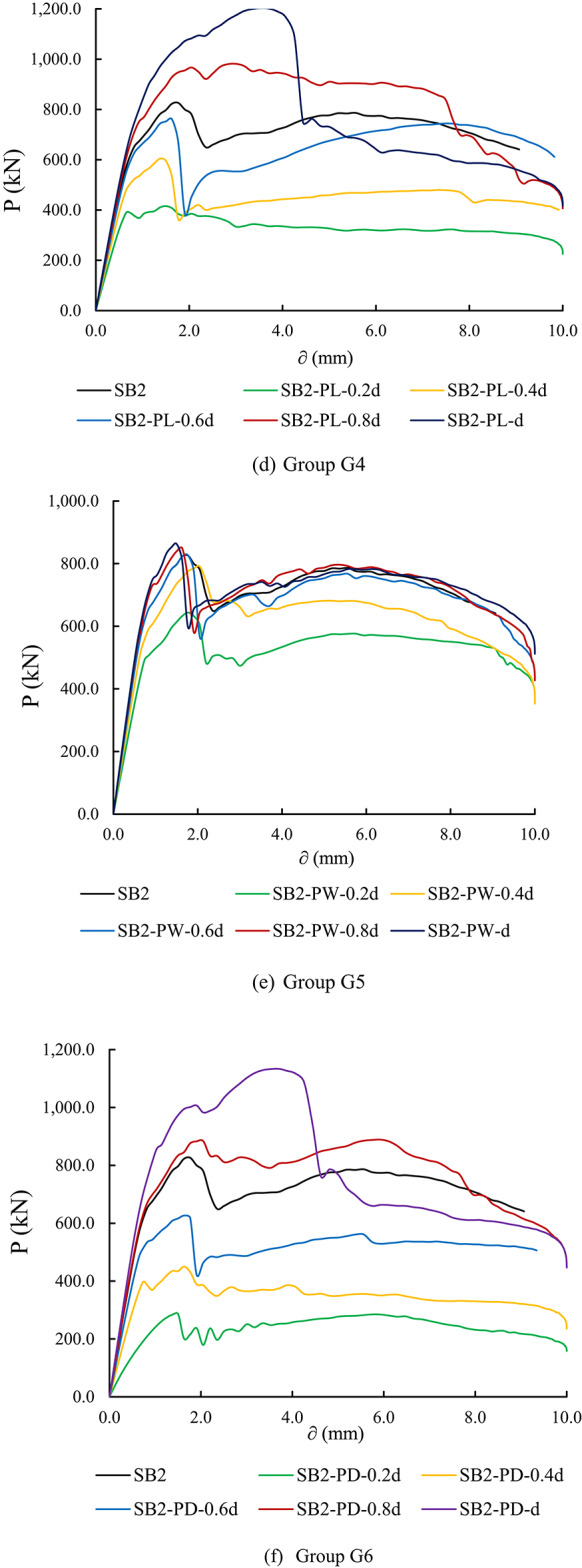
Load- deflection curves of parametric analysis specimens.

Figure 17 illustrates the load versus mid-span deflection (P-δ) curves for the parametric analysis specimens at different loading stages. Overall, it is evident that variations in the configuration (rectangular and circular) and size of the columns and piles significantly influenced the P-δ relationship at all loading stages for the tested specimens.

The Group G1 specimens showed that increasing the rectangular column length from 0.2 d to d improves the P-δ relationship. Additionally, the elastic stiffness of the tested specimens generally increased, as evidenced by the slope of the load-deflection curve. Specimen SB2-CL-d, with a rectangular column length of d (where d represents the pile cap width), exhibited the best P-δ relationship, with improvements in both pile cap stiffness and ultimate load capacity compared to the control specimen (Fig. 17a).

Similarly, Group G2 specimens demonstrated a significant improvement in the load-deflection relationship of the pile cap as the rectangular column width increased, accompanied by enhanced elastic stiffness. Specimen SB2-CW-d showed the best performance in terms of ultimate load capacity and pile cap stiffness. Additionally, the behavior of specimens SB2-CW-0.6 d, SB2-CW-0.8 d, and SB2-CW-d was found to be similar, indicating that increasing the rectangular column width beyond 0.6 d does not notably affect the load-deflection relationship or the stiffness of the pile cap (Fig. 17b).

Additionally, in Group G3, which investigates the impact of circular columns on pile cap performance, the results indicate that increasing the circular column diameter enhances both the P-δ relationship and the stiffness of the pile cap. As depicted in Fig. 17c, a significant influence of column diameter on the P-δ relationship of the tested specimens is evident, particularly in comparison to the specimens with rectangular columns.

To evaluate the impact of rectangular pile dimensions on the P-δ relationship of pile caps, Group G4 specimens, which explore the effects of varying rectangular pile lengths, demonstrate that increasing the length of the piles leads to significant improvements in both the P-δ behavior and stiffness of the pile cap. As illustrated in Fig. 17d, rectangular pile length plays a crucial role in enhancing the performance of the tested pile caps. In contrast, for Group G5, although increasing the rectangular pile width results in some improvement in both the P-δ behavior and pile cap stiffness, the effect is more modest (Fig. 17e), similar to the behavior observed in Group G2 specimens. On the other hand, a notable improvement is observed in the load-deflection relationship and stiffness of pile caps for Group G6 specimens as the pile diameter increases (Fig. 17f), with this effect being as pronounced as that observed in Group G3 specimens.

#### Ultimate loads

**Fig. 18:**
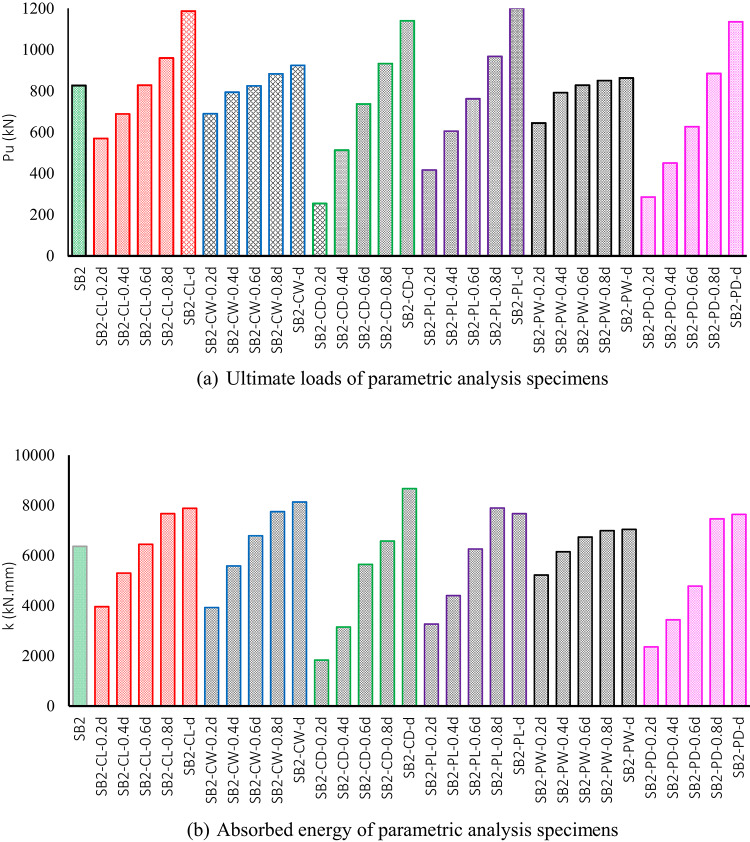
Ultimate loads and absorbed energy of parametric analysis specimens.

Table 8 displays the ultimate load ($\:{P}_{u}$) recorded for parametric analysis specimens, illustrating the impact of different column and pile configuration and size on their structural performance. In general, increasing the size of columns and piles enhances the ultimate load capacity, as shown in Fig. 18a.

In Group G1, which examines the influence of rectangular column length on $\:{P}_{u}$ of pile cap, the results indicate ultimate loads of 569 kN, 668 kN, 827 kN, 959 kN, and 1186 kN for specimens SB2-CL-0.2 d, SB2-CL-0.4 d, SB2-CL-0.6 d, SB2-CL-0.8 d, and SB2-CL-d, respectively, as outlined in Table 8. These values demonstrate that increasing the rectangular column length enhances the ultimate load capacity. A substantial improvement of 108% is observed as the rectangular column length increases from 0.2 d to d, where **d** represents the pile cap width. Additionally, the ultimate load increases by 16% and 44% for rectangular column lengths of 0.8 d and d, respectively, compared to the control pile cap (SB2). These results further emphasize the crucial role of increasing the rectangular column length in improving the ultimate load capacity, as illustrated in Fig. 18a. The observed improvements in ultimate load capacity with increased rectangular column length can be attributed to the enhanced distribution of applied loads and shear forces across the pile cap. As the rectangular column length increases, the load is more effectively transferred to the piles, leading to a better resistance against shear and bending stresses. This increased length also provides greater structural stiffness, reducing deformation and improving the overall load-bearing capacity of the pile cap.

In Group G2, which examines the effect of rectangular column width on the ultimate load ($\:{P}_{u}$) of the pile cap, the results indicate ultimate loads of 689 kN, 793 kN, 824 kN, 882 kN, and 923 kN for specimens SB2-CW-0.2 d, SB2-CW-0.4 d, SB2-CW-0.6 d, SB2-CW-0.8 d, and SB2-CW-d, respectively, as shown in Table 8. These results demonstrate that increasing the rectangular column width enhances the ultimate load capacity, with a significant 34% improvement when the rectangular column width increases from 0.2 d to d. Additionally, the ultimate load increases by 7% and 12% for rectangular column widths of 0.8 d and d, respectively, compared to the control pile cap (SB2). However, although the ultimate load increases with rectangular column width, the overall enhancement is relatively modest, suggesting that the influence of column width on ultimate load capacity is less significant than that of rectangular column length, as shown in Fig. 18a.

Additionally, in Group G3, which examines the effect of circular column on the ultimate load ($\:{P}_{u}$) of the pile cap, the results indicate ultimate loads of 254 kN, 513 kN, 736 kN, 932 kN, and 1139 kN for specimens SB2-CD-0.2 d, SB2-CD-0.4 d, SB2-CD-0.6 d, SB2-CD-0.8 d, and SB2-CD-d, respectively, as shown in Table 8. These results demonstrate that increasing the circular column diameter enhances the ultimate load capacity, with a significant 348% improvement when the circular column diameter increases from 0.2 d to d. Additionally, the ultimate load increases by 13% and 38% for circular column diameters of 0.8 d and d, respectively, compared to the control pile cap (SB2). These results further emphasize the crucial role of increasing the circular column diameter in improving the ultimate load capacity. Moreover, the comparison between rectangular and circular columns demonstrates that rectangular columns are more efficient in enhancing the ultimate load capacity, particularly with dimensions ranging from 0.2 d to 0.6 d, as illustrated in Fig. 18a.

To explore the effect of rectangular pile configurations and sizes on ultimate load capacity, Group G4 specimens, which focus on the influence of rectangular pile length on $\:{P}_{u}$, reveal that an increase in rectangular pile length results in a corresponding increase in ultimate load. The results show ultimate loads of 416 kN, 604 kN, 761 kN, 967 kN, and 1202 kN for specimens SB2-CL-0.2 d, SB2-CL-0.4 d, SB2-CL-0.6 d, SB2-CL-0.8 d, and SB2-CL-d, respectively, as detailed in Table 8. A substantial improvement of 188% is observed as the rectangular pile length increases from 0.2 d to d. Additionally, the ultimate load increases by 17% and 46% for pile lengths of 0.8 d and d, respectively, compared to the control pile cap (SB2). These results further emphasize the crucial role of increasing the rectangular pile length in improving the ultimate load capacity, as illustrated in Fig. 18a.

Additionally, Group G5 specimens, which investigate the effect of rectangular pile width on $\:{P}_{u}$, demonstrate that increasing the width of rectangular piles leads to a corresponding rise in ultimate load. The ultimate load capacities for specimens SB2-PW-0.2 d, SB2-PW-0.4 d, SB2-PW-0.6 d, SB2-PW-0.8 d, and SB2-PW-d are 644 kN, 791 kN, 827 kN, 849 kN, and 862 kN, respectively. These findings show that increasing the rectangular pile width enhances the ultimate load capacity, with a significant 34% increase when the pile width grows from 0.2 d to d. Moreover, the ultimate load increases by 3% and 4% for pile widths of 0.8 d and d, respectively, compared to the control pile cap (SB2). The results also demonstrate that any increase in pile width beyond 0.6 d has a negligible impact. Therefore, it is advisable to avoid exceeding a pile width of 0.6 d.

In Group G6, which examines the effect of circular pile on the ultimate load ($\:{P}_{u}$) of the pile cap, the results indicate ultimate loads of 285 kN, 450 kN, 626 kN, 884 kN, and 1134 kN for specimens SB2-PD-0.2 d, SB2-PD-0.4 d, SB2-PD-0.6 d, SB2-PD-0.8 d, and SB2-PD-d, respectively, as shown in Table 8. These results demonstrate that increasing the circular pile diameter enhances the ultimate load capacity, with a significant 297% improvement when the circular column diameter increases from 0.2 d to d. Additionally, the ultimate load increases by 7% and 37% for circular pile diameters of 0.8 d and d, respectively, compared to the control pile cap (SB2). These results further emphasize the crucial role of increasing the circular pile diameter in improving the ultimate load capacity. Moreover, the comparison between rectangular and circular piles demonstrates that rectangular piles are more efficient in enhancing the ultimate load capacity, particularly with dimensions ranging from 0.2 d to 0.6 d, as illustrated in Fig. 18a.

In comparison between the changes in the configurations (rectangular or circular) and sizes of columns and piles, it is evident that there is a strong similarity in ultimate load capacity values (Fig. 18a). Therefore, altering the configuration or size of columns or piles has a similar effect on the ultimate load capacity of the pile cap. Additionally, the specimen SB2-PL-d, which features a rectangular pile configuration with dimensions (d × 0.6 d), ranked first in terms of the highest ultimate load capacity of the pile cap. It was followed by the specimens SB2-CL-d, SB2-CD-d, and SB2-PD-d. Meanwhile, the SB2-CD-0.2 d, which features a circular column with a 0.2 d diameter, ranked last.

#### Energy absorption

Table 8 shows the computed values of energy absorption for parametric analysis specimens. absorbed energy (E) evaluates the beam’s ability to withstand and dissipate energy under applied loads. Overall, increasing the size of columns and piles enhances the energy absorption, as shown in Fig. 18b.

As outlined in Table 8, the Group G1 specimens demonstrated that increasing the rectangular column length from 0.2 d to d enhances the absorbed energy by 100%. Additionally, the absorbed energy increases by 20% and 24% for rectangular column lengths of 0.8 d and d, respectively, compared to the control pile cap (SB2). These findings highlight the significant role of increasing the rectangular column length in enhancing absorbed energy capacity, as illustrated in Fig. 18b.

Furthermore, the specimens of Group G2 exhibited a comparable improvement to those in Group G1, with a 107% increase in absorbed energy when the rectangular column width increased from 0.2 d to d. Additionally, specimens SB2-CW-0.8 d and SB2-CW-d, with widths of 0.8 d and d, showed further increases of 22% and 28%, respectively. These findings suggest that modifying either the rectangular column length or width has a similar impact on the energy absorption capacity of the pile cap.

In Group G3, the results indicate a 373% increase in absorbed energy as the circular column diameter increases from 0.2 d to d. Additionally, specimen SB2-CD-d, with a diameter of d, exhibited a notable 36% increase compared to the control specimen (SB2), making it the best-performing specimen within the group. These findings further highlight the significant role of increasing the circular column diameter in enhancing the energy absorption capacity of the pile cap.

In addition, the specimens of Group G4 exhibited a 142% increase in absorbed energy as the rectangular pile length increased from 0.2 d to 0.8 d. Furthermore, specimen SB2-PL-0.8 d, featuring a rectangular pile with a length of 0.8 d, showed the best performance within the group, with a 24% improvement compared to the control specimen. However, the results also showed that increasing the rectangular pile length to d (pile cap width) resulted in a decrease in the pile cap’s energy absorption capacity, despite this specimen (SB2-PL-d) achieving the highest ultimate load capacity among all the tested specimens.

In addition, the specimens of Group G4 demonstrated a 142% increase in absorbed energy as the rectangular pile length increased from 0.2 d to 0.8 d. Furthermore, specimen SB2-PL-0.8 d, featuring a rectangular pile with a length of 0.8 d, showed the best performance within the group, with a 24% improvement compared to the control specimen. However, specimens SB2-CD-d exhibited only a 20% increase in absorbed energy, suggesting that increasing the rectangular pile length to d (as in specimen SB2-CD-d) led to a decrease in the pile cap’s energy absorption capacity, despite SB2-CD-d achieving the highest ultimate load capacity among all the tested specimens. In Group G5, a 34% increase in absorbed energy was observed as the rectangular pile width increased from 0.2 d to d. Furthermore, specimens SB2-PW-0.8 d and SB2-PW-d exhibited the same absorbed energy, indicating that increasing the rectangular pile width beyond 0.8 d does not significantly affect the energy absorption capacity of the beams.

Similarly, the results indicated a 224% improvement in the absorbed energy of Group G6 specimens as the circular pile diameter increased from 0.2 d to d. Furthermore, specimens SB2-CD-0.8 d and SB2-CD-d exhibited nearly the same absorbed energy, suggesting that increasing the circular pile diameter beyond 0.8 d does not significantly impact the energy absorption capacity of the beams.

Among the parametric analysis specimens, SB2-CD-d, featuring a circular column with a diameter equal to d, demonstrated the highest effectiveness in improving the absorbed energy of the pile cap, as shown in Fig. 17b. This was followed by specimens SB2-CW-d, SB2-CL-d, SB2-PL-0.8 d, and SB2-CL-d. In contrast, the specimens SB2-CD-0.2 d, with a circular column diameter of 0.2 d, ranked last.

**Table 8:** Outcomes of parametric analysis specimens.

Group	Specimen`s ID	Ultimate load		Energy absorption	
		*P*_*u*_ (kN)	*P*_*u*_ */P*_*u, o*_	*E* (kN.mm)	*E/E*_*o*_
G1	SB2	826	1.00	6367.70	1.00
	SB2-CL-0.2 d	569	0.69	3963.50	0.62
	SB2-CL-0.4 d	688	0.83	5300.50	0.83
	SB2-CL-0.6 d	827	1.00	6445.74	1.01
	SB2-CL-0.8 d	959	1.16	7670.50	1.20
	SB2-CL-d	1186	1.44	7883.30	1.24
G2	SB2	826	1.00	6367.70	1.00
	SB2-CW-0.2 d	689	0.83	3926.7	0.62
	SB2-CW-0.4 d	793	0.96	5585.08	0.88
	SB2-CW-0.6 d	824	1.00	6787.30	1.07
	SB2-CW-0.8 d	882	1.07	7748.50	1.22
	SB2-CW-d	923	1.12	8135.24	1.28
G3	SB2	826	1.00	6367.70	1.00
	SB2-CD-0.2 d	254	0.31	1831.60	0.29
	SB2-CD-0.4 d	513	0.62	3149.30	0.49
	SB2-CD-0.6 d	736	0.89	5647.53	0.89
	SB2-CD-0.8 d	932	1.13	6575.04	1.03
	SB2-CD-d	1139	1.38	8666.13	1.36
G4	SB2	826	1.00	6367.70	1.00
	SB2-PL-0.2 d	416	0.50	3267.30	0.51
	SB2-PL-0.4 d	604	0.73	4401.40	0.69
	SB2-PL-0.6 d	761	0.92	6260.40	0.98
	SB2-PL-0.8 d	967	1.17	7893.22	1.24
	SB2-PL-d	1202	1.46	7669.61	1.20
G5	SB2	826	1.00	6367.70	1.00
	SB2-PW-0.2 d	644	0.78	5224.50	0.82
	SB2-PW-0.4 d	791	0.96	6147.87	0.97
	SB2-PW-0.6 d	827	1.00	6736.87	1.06
	SB2-PW-0.8 d	849	1.03	6990.67	1.10
	SB2-PW-d	862	1.04	7040.45	1.11
G6	SB2	826	1.00	6367.70	1.00
	SB2-PD-0.2 d	285	0.35	2357	0.37
	SB2-PD-0.4 d	450	0.54	3438.50	0.54
	SB2-PD-0.6 d	626	0.76	4780.90	0.75
	SB2-PD-0.8 d	884	1.07	7465.60	1.17
	SB2-PD-d	1134	1.37	7639.30	1.20

$\:{P}_{u}$: Ultimate load of the specimen,$\:{P}_{uo}$: Ultimate load of specimen SB2, E: Absorbed energy of the specimen, and *E*_*o*_ : Absorbed energy of the specimen SB2. .

## Conclusion

This study investigated the shear performance of reinforced concrete (RC) pile caps through experimental and numerical analyses, focusing on the influence of column and pile configurations and dimensions. Two pile cap specimens (SB1 and SB2) with varying heights (250 mm and 350 mm) were tested under shear-dominated conditions, supported by square piles and loaded via a square column. A validated 3D finite element model was developed to parametrically analyze rectangular and circular column/pile configurations with dimensions ranging from 0.2 d to d (where d = pile cap width). For all evaluated pile caps, the research also presents results on failure patterns, load-deflection behavior, ultimate load capacity, and energy absorption capacity. The results showed the following:

1. All specimens exhibited shear-dominated failure, with diagonal cracks propagating between the column and piles.
2. Variations in column/pile configuration and size did not alter the failure mechanism.
3. Increasing rectangular column length from 0.2 d to d enhanced ultimate load capacity by 108%.
4. Circular column diameter increases (0.2 d to d) improved load capacity by 348%, outperforming rectangular configurations.
5. Larger pile dimensions (rectangular or circular) also increased load capacity, with rectangular pile length showing a 188% improvement.
6. Circular columns demonstrated superior energy absorption (373% increase for diameter 0.2 d to d).
7. Rectangular column width and pile length expansions improved energy dissipation by 107% and 142%, respectively.
8. Circular columns/piles offer greater shear performance enhancements compared to rectangular ones.
9. Optimal performance is achieved when column/pile dimensions approach the pile cap width (d).

The results provide actionable insights for optimizing pile cap design, emphasizing the benefits of larger column/pile dimensions and circular configurations to enhance shear resistance and energy absorption. These findings align with strut-and-tie model principles and can inform code-based design practices for RC pile caps.

## Data Availability

The datasets used and/or analyzed during the current study available from the corresponding author on reasonable request.
